# 4-Chloro-*N*-(3-methyl­phen­yl)benzamide

**DOI:** 10.1107/S1600536811040864

**Published:** 2011-10-08

**Authors:** Vinola Z. Rodrigues, Marek Fronc, B. Thimme Gowda, Jozef Kožíšek

**Affiliations:** a Department of Chemistry, Mangalore University, Mangalagangotri 574 199, Mangalore, India; b Institute of Physical Chemistry and Chemical Physics, Slovak University of Technology, Radlinského 9, SK-812 37 Bratislava, Slovak Republic

## Abstract

In the title compound, C_14_H_12_ClNO, the *meta*-methyl substituent in the aniline ring is positioned *anti* to the N—H bond. The dihedral angle between the rings is 12.4 (1)°. The crystal structure is stabilized by inter­molecular N—H⋯O hydrogen bonds, which link the mol­ecules into *C*(4) chains running along the *c*-axis direction.

## Related literature

For the preparation of the title compound, see: Gowda *et al.* (2003 ▶). For studies on the effects of substituents on the structures and other aspects of *N*-(ar­yl)-amides, see: Bowes *et al.* (2003 ▶); Gowda *et al.* (2000 ▶); Saeed *et al.* (2010 ▶), on *N*-(ar­yl)-methane­sulfonamides, see: Gowda *et al.* (2007 ▶), on *N*-(ar­yl)-aryl­sulfonamides, see: Shetty & Gowda (2005 ▶) and on *N*-chloro-aryl­sulfonamides, see: Gowda & Shetty (2004 ▶).
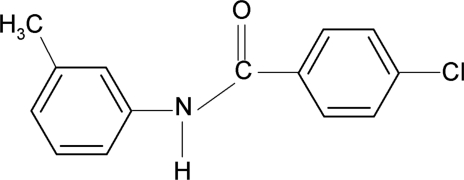


## Experimental

### 

#### Crystal data

C_14_H_12_ClNO
*M* *_r_* = 245.70
Monoclinic, 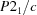

*a* = 13.4379 (9) Å
*b* = 10.2493 (11) Å
*c* = 9.2600 (7) Å
β = 92.893 (6)°
*V* = 1273.74 (19) Å^3^
*Z* = 4
Mo *K*α radiation
μ = 0.28 mm^−1^
*T* = 293 K
0.81 × 0.17 × 0.04 mm

#### Data collection

Oxford Diffraction Xcalibur diffractometer with a Ruby (Gemini Cu) detector
Absorption correction: analytical [*CrysAlis RED* (Oxford Diffraction, 2009 ▶), based on expressions derived from Clark & Reid (1995 ▶)] *T*_min_ = 0.877, *T*_max_ = 0.988
17187 measured reflections
2156 independent reflections
1299 reflections with *I* > 2σ(*I*)
*R*_int_ = 0.040

#### Refinement

*R*[*F* ^2^ > 2σ(*F* ^2^)] = 0.040
*wR*(*F* ^2^) = 0.117
*S* = 0.94
2156 reflections
154 parameters
H-atom parameters constrained
Δρ_max_ = 0.22 e Å^−3^
Δρ_min_ = −0.13 e Å^−3^

### 

Data collection: *CrysAlis CCD* (Oxford Diffraction, 2009 ▶); cell refinement: *CrysAlis CCD*; data reduction: *CrysAlis RED* (Oxford Diffraction, 2009 ▶); program(s) used to solve structure: *SHELXS97* (Sheldrick, 2008 ▶); program(s) used to refine structure: *SHELXL97* (Sheldrick, 2008 ▶); molecular graphics: *Mercury* (Macrae *et al.*, 2008 ▶); software used to prepare material for publication: *enCIFer* (Allen *et al.*, 2004 ▶).

## Supplementary Material

Crystal structure: contains datablock(s) I, global. DOI: 10.1107/S1600536811040864/bt5661sup1.cif

Structure factors: contains datablock(s) I. DOI: 10.1107/S1600536811040864/bt5661Isup2.hkl

Supplementary material file. DOI: 10.1107/S1600536811040864/bt5661Isup3.cml

Additional supplementary materials:  crystallographic information; 3D view; checkCIF report

## Figures and Tables

**Table 1:** Hydrogen-bond geometry (Å, °)

*D*—H⋯*A*	*D*—H	H⋯*A*	*D*⋯*A*	*D*—H⋯*A*
N1—H1*A*⋯O1^i^	0.86	2.06	2.888 (2)	161

Symmetry code: (i) 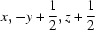.

**Fig. 1.**
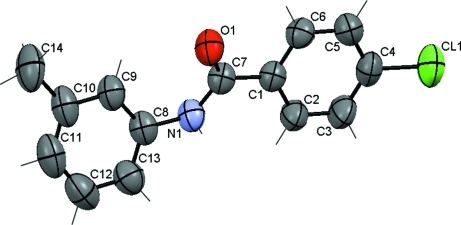
Molecular structure of the title compound showing the atom labelling scheme. Displacement ellipsoids are drawn at the 50% probability level. H atoms are represented as small spheres of arbitrary radii.

**Fig. 2.**
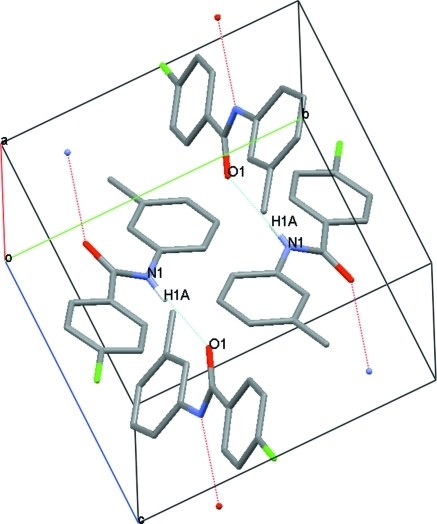
Part of the crystal structure of the title compound generated by N—H···O hydrogen bonds which are shown by dashed lines. H atoms not involved in intermolecular bonding have been omitted.

## supplementary crystallographic information

### Comment

The amide and sulfonamide moieties are the constituents of many biologically important compounds. As part of our studies on the substituent effects on the structures and other aspects of *N*-(aryl)-amides (Bowes *et al.*, 2003; Gowda *et al.*, 2000; Saeed *et al.*, 2010), *N*-(aryl)-methanesulfonamides (Gowda *et al.*, 2007), *N*-(aryl)-arylsulfonamides (Shetty & Gowda, 2005) and *N*-chloro-arylsulfonamides (Gowda & Shetty, 2004), in the present work, the crystal structure of 4-chloro-*N*-(3-methylphenyl)benzamide (I) has been determined (Fig.1). In (I), the *meta*-methyl substituent in the anilino ring is positioned *anti* to the N–H bond.

The central amide group –NHCO– is tilted to the anilino ring with the C9—C8—N1—C7 and C13—C8—N1—C7 torsion angles of 30.4 (3)° and -151.2 (2)°. The C2—C1—C7—N1 and C6—C1—C7—N1 torsion angles are -15.7 (3)° and 166.8 (2)°, respectively, while the C2—C1—C7—O1 and C6—C1—C7—O1 torsion angles are 162.8 (2)° and -14.7 (3)°, respectively. But the C1—C7—N1—C8 and C8—N1—C7—O1 torsion angles are 173.6 (2)° and -4.9 (3)°, respectively.

The packing of molecules linked by N—H···O hydrogen bonds is shown in Fig. 2.

### Experimental

The title compound was prepared according to the method described by Gowda *et al.* (2003). The purity of the compound was checked by determining its melting point. It was characterized by recording its infrared and NMR spectra. Plate like colourless single crystals of the title compound were obtained by slow evaporation of an ethanol solution of the compound (0.5 g in about 30 ml of ethanol) at room temperature.

### Refinement

All H atoms were visible in difference maps and then treated as riding atoms with C–H distances of 0.93Å (C-aromatic), 0.96Å (*C*-methyl) and N—H = 0.86 Å. The *U*_iso_(H) values were set at 1.2 *U*_eq_(C-aromatic, N) and 1.5 *U*_eq_(*C*-methyl).

### Crystal data

**Table:**

C_14_H_12_ClNO	*F*(000) = 512
*M**_r_* = 245.70	*D*_x_ = 1.281 Mg m^−^^3^
Monoclinic, *P*2_1_/*c*	Mo *K*α radiation, λ = 0.71073 Å
Hall symbol: -p 2ybc	Cell parameters from 4939 reflections
*a* = 13.4379 (9) Å	θ = 3.4–29.4°
*b* = 10.2493 (11) Å	µ = 0.28 mm^−^^1^
*c* = 9.2600 (7) Å	*T* = 293 K
β = 92.893 (6)°	Plate, colorless
*V* = 1273.74 (19) Å^3^	0.81 × 0.17 × 0.04 mm
*Z* = 4	

### Data collection

**Table:**

Oxford Diffraction Xcalibur diffractometer with a Ruby (Gemini Cu) detector	2156 independent reflections
Radiation source: fine-focus sealed tube	1299 reflections with *I* > 2σ(*I*)
graphite	*R*_int_ = 0.040
ω scans	θ_max_ = 24.7°, θ_min_ = 4.2°
Absorption correction: analytical [*CrysAlis RED* (Oxford Diffraction, 2009), based on expressions derived from Clark & Reid (1995)]	*h* = −15→15
*T*_min_ = 0.877, *T*_max_ = 0.988	*k* = −12→12
17187 measured reflections	*l* = −10→10

### Refinement

**Table:**

Refinement on *F*^2^	Primary atom site location: structure-invariant direct methods
Least-squares matrix: full	Secondary atom site location: difference Fourier map
*R*[*F*^2^ > 2σ(*F*^2^)] = 0.040	Hydrogen site location: inferred from neighbouring sites
*wR*(*F*^2^) = 0.117	H-atom parameters constrained
*S* = 0.94	*w* = 1/[σ^2^(*F*_o_^2^) + (0.0699*P*)^2^] where *P* = (*F*_o_^2^ + 2*F*_c_^2^)/3
2156 reflections	(Δ/σ)_max_ < 0.001
154 parameters	Δρ_max_ = 0.22 e Å^−^^3^
0 restraints	Δρ_min_ = −0.13 e Å^−^^3^

### Special details

**Table:**

Experimental. CrysAlis RED (Oxford Diffraction, 2009) Analytical numeric absorption correction using a multifaceted crystal model based on expressions derived (Clark & Reid, 1995).
Geometry. All e.s.d.'s (except the e.s.d. in the dihedral angle between two l.s. planes) are estimated using the full covariance matrix. The cell e.s.d.'s are taken into account individually in the estimation of e.s.d.'s in distances, angles and torsion angles; correlations between e.s.d.'s in cell parameters are only used when they are defined by crystal symmetry. An approximate (isotropic) treatment of cell e.s.d.'s is used for estimating e.s.d.'s involving l.s. planes.
Refinement. Refinement of *F*^2^ against ALL reflections. The weighted *R*-factor *wR* and goodness of fit *S* are based on *F*^2^, conventional *R*-factors *R* are based on *F*, with *F* set to zero for negative *F*^2^. The threshold expression of *F*^2^ > σ(*F*^2^) is used only for calculating *R*-factors(gt) *etc*. and is not relevant to the choice of reflections for refinement. *R*-factors based on *F*^2^ are statistically about twice as large as those based on *F*, and *R*- factors based on ALL data will be even larger.

### Fractional atomic coordinates and isotropic or equivalent isotropic displacement parameters (Å^2^)

**Table:**

	*x*	*y*	*z*	*U*_iso_*/*U*_eq_	
C1	0.44789 (14)	0.3375 (2)	−0.05692 (18)	0.0570 (5)	
C2	0.40134 (16)	0.2716 (2)	0.0505 (2)	0.0749 (6)	
H2A	0.4350	0.2045	0.0999	0.090*	
C3	0.30600 (17)	0.3031 (3)	0.0860 (3)	0.0850 (7)	
H3A	0.2759	0.2590	0.1600	0.102*	
C4	0.25583 (15)	0.4008 (3)	0.0105 (3)	0.0746 (6)	
C5	0.30042 (16)	0.4691 (2)	−0.0950 (2)	0.0715 (6)	
H5A	0.2665	0.5361	−0.1441	0.086*	
C6	0.39659 (15)	0.4374 (2)	−0.1280 (2)	0.0651 (6)	
H6A	0.4274	0.4841	−0.1994	0.078*	
C7	0.54954 (14)	0.3041 (2)	−0.10552 (19)	0.0587 (5)	
C8	0.70086 (15)	0.1721 (2)	−0.0475 (2)	0.0636 (6)	
C9	0.76789 (15)	0.2328 (2)	−0.1344 (2)	0.0695 (6)	
H9A	0.7512	0.3126	−0.1768	0.083*	
C10	0.85939 (16)	0.1769 (3)	−0.1594 (2)	0.0806 (7)	
C11	0.88194 (19)	0.0584 (3)	−0.0961 (3)	0.0932 (8)	
H11A	0.9423	0.0185	−0.1139	0.112*	
C12	0.8172 (2)	−0.0028 (3)	−0.0066 (3)	0.0966 (8)	
H12A	0.8347	−0.0820	0.0366	0.116*	
C13	0.72518 (18)	0.0546 (3)	0.0188 (2)	0.0811 (7)	
H13A	0.6812	0.0144	0.0793	0.097*	
C14	0.93185 (19)	0.2429 (3)	−0.2533 (3)	0.1117 (10)	
H14C	0.9906	0.1900	−0.2584	0.134*	
H14B	0.9496	0.3265	−0.2129	0.134*	
H14A	0.9018	0.2544	−0.3487	0.134*	
N1	0.60639 (12)	0.22675 (17)	−0.01846 (16)	0.0636 (5)	
H1A	0.5833	0.2081	0.0640	0.076*	
O1	0.57677 (10)	0.34502 (16)	−0.22211 (13)	0.0762 (5)	
Cl1	0.13343 (4)	0.43547 (8)	0.04942 (9)	0.1151 (4)	

### Atomic displacement parameters (Å^2^)

**Table:**

	*U*^11^	*U*^22^	*U*^33^	*U*^12^	*U*^13^	*U*^23^
C1	0.0537 (11)	0.0700 (14)	0.0472 (10)	−0.0039 (11)	0.0032 (8)	−0.0056 (10)
C2	0.0575 (13)	0.0910 (17)	0.0767 (14)	0.0007 (11)	0.0087 (11)	0.0183 (12)
C3	0.0605 (14)	0.108 (2)	0.0887 (16)	−0.0038 (14)	0.0215 (12)	0.0212 (14)
C4	0.0506 (12)	0.0875 (17)	0.0864 (14)	−0.0042 (11)	0.0101 (11)	−0.0100 (13)
C5	0.0633 (13)	0.0758 (16)	0.0751 (13)	0.0058 (11)	0.0019 (11)	−0.0011 (11)
C6	0.0650 (13)	0.0747 (15)	0.0562 (11)	−0.0025 (11)	0.0096 (10)	−0.0003 (10)
C7	0.0562 (12)	0.0761 (14)	0.0438 (10)	−0.0039 (10)	0.0031 (9)	−0.0071 (9)
C8	0.0598 (12)	0.0813 (16)	0.0498 (10)	0.0084 (11)	0.0022 (9)	−0.0116 (11)
C9	0.0598 (13)	0.0931 (17)	0.0558 (11)	0.0087 (12)	0.0043 (10)	−0.0080 (10)
C10	0.0590 (14)	0.123 (2)	0.0590 (12)	0.0198 (14)	−0.0026 (10)	−0.0145 (13)
C11	0.0670 (15)	0.131 (3)	0.0807 (15)	0.0254 (16)	−0.0042 (13)	−0.0256 (17)
C12	0.091 (2)	0.095 (2)	0.1014 (19)	0.0246 (16)	−0.0190 (16)	−0.0062 (15)
C13	0.0794 (16)	0.0897 (19)	0.0739 (14)	0.0095 (14)	0.0015 (12)	−0.0021 (13)
C14	0.0646 (15)	0.179 (3)	0.0930 (17)	0.0155 (17)	0.0231 (13)	−0.0068 (17)
N1	0.0575 (10)	0.0846 (13)	0.0494 (9)	0.0063 (9)	0.0104 (7)	−0.0001 (8)
O1	0.0624 (8)	0.1183 (13)	0.0485 (8)	0.0053 (8)	0.0093 (6)	0.0064 (8)
Cl1	0.0586 (4)	0.1374 (7)	0.1517 (7)	0.0078 (4)	0.0288 (4)	0.0031 (5)

### Geometric parameters (Å, °)

**Table:**

C1—C2	1.378 (3)	C8—C9	1.386 (3)
C1—C6	1.382 (3)	C8—N1	1.426 (3)
C1—C7	1.499 (3)	C9—C10	1.387 (3)
C2—C3	1.377 (3)	C9—H9A	0.9300
C2—H2A	0.9300	C10—C11	1.375 (4)
C3—C4	1.378 (3)	C10—C14	1.499 (4)
C3—H3A	0.9300	C11—C12	1.382 (4)
C4—C5	1.365 (3)	C11—H11A	0.9300
C4—Cl1	1.738 (2)	C12—C13	1.400 (4)
C5—C6	1.381 (3)	C12—H12A	0.9300
C5—H5A	0.9300	C13—H13A	0.9300
C6—H6A	0.9300	C14—H14C	0.9600
C7—O1	1.231 (2)	C14—H14B	0.9600
C7—N1	1.342 (2)	C14—H14A	0.9600
C8—C13	1.383 (3)	N1—H1A	0.8600
			
C2—C1—C6	118.24 (19)	C8—C9—C10	121.3 (2)
C2—C1—C7	123.92 (19)	C8—C9—H9A	119.4
C6—C1—C7	117.79 (17)	C10—C9—H9A	119.4
C3—C2—C1	121.3 (2)	C11—C10—C9	118.2 (2)
C3—C2—H2A	119.4	C11—C10—C14	120.7 (2)
C1—C2—H2A	119.4	C9—C10—C14	121.2 (3)
C2—C3—C4	119.1 (2)	C10—C11—C12	121.6 (2)
C2—C3—H3A	120.4	C10—C11—H11A	119.2
C4—C3—H3A	120.4	C12—C11—H11A	119.2
C5—C4—C3	121.0 (2)	C11—C12—C13	119.9 (3)
C5—C4—Cl1	119.86 (19)	C11—C12—H12A	120.0
C3—C4—Cl1	119.17 (18)	C13—C12—H12A	120.0
C4—C5—C6	119.1 (2)	C8—C13—C12	118.8 (2)
C4—C5—H5A	120.4	C8—C13—H13A	120.6
C6—C5—H5A	120.4	C12—C13—H13A	120.6
C5—C6—C1	121.23 (19)	C10—C14—H14C	109.5
C5—C6—H6A	119.4	C10—C14—H14B	109.5
C1—C6—H6A	119.4	H14C—C14—H14B	109.5
O1—C7—N1	122.88 (18)	C10—C14—H14A	109.5
O1—C7—C1	120.08 (17)	H14C—C14—H14A	109.5
N1—C7—C1	117.03 (16)	H14B—C14—H14A	109.5
C13—C8—C9	120.2 (2)	C7—N1—C8	127.20 (16)
C13—C8—N1	116.8 (2)	C7—N1—H1A	116.4
C9—C8—N1	123.0 (2)	C8—N1—H1A	116.4
			
C6—C1—C2—C3	0.4 (3)	C13—C8—C9—C10	1.3 (3)
C7—C1—C2—C3	−177.07 (19)	N1—C8—C9—C10	179.60 (17)
C1—C2—C3—C4	1.3 (4)	C8—C9—C10—C11	0.4 (3)
C2—C3—C4—C5	−2.2 (4)	C8—C9—C10—C14	−179.9 (2)
C2—C3—C4—Cl1	176.97 (18)	C9—C10—C11—C12	−1.7 (3)
C3—C4—C5—C6	1.3 (3)	C14—C10—C11—C12	178.6 (2)
Cl1—C4—C5—C6	−177.83 (15)	C10—C11—C12—C13	1.4 (4)
C4—C5—C6—C1	0.5 (3)	C9—C8—C13—C12	−1.6 (3)
C2—C1—C6—C5	−1.3 (3)	N1—C8—C13—C12	179.96 (18)
C7—C1—C6—C5	176.33 (17)	C11—C12—C13—C8	0.3 (3)
C2—C1—C7—O1	162.80 (19)	O1—C7—N1—C8	−4.9 (3)
C6—C1—C7—O1	−14.7 (3)	C1—C7—N1—C8	173.59 (17)
C2—C1—C7—N1	−15.7 (3)	C13—C8—N1—C7	−151.2 (2)
C6—C1—C7—N1	166.77 (17)	C9—C8—N1—C7	30.4 (3)

### Hydrogen-bond geometry (Å, °)

**Table:**

*D*—H···*A*	*D*—H	H···*A*	*D*···*A*	*D*—H···*A*
N1—H1A···O1^i^	0.86	2.06	2.888 (2)	161.

Symmetry codes: (i) *x*, −*y*+1/2, *z*+1/2.
